# Edge Detection in Landing Budgerigars (*Melopsittacus undulatus*)

**DOI:** 10.1371/journal.pone.0007301

**Published:** 2009-10-07

**Authors:** Partha Bhagavatula, Charles Claudianos, Michael Ibbotson, Mandyam Srinivasan

**Affiliations:** 1 ARC Centre of Excellence in Vision Science, Australian National University, Canberra, Australian Capital Territory, Australia; 2 Research School of Biological Sciences, Australian National University, Canberra, Australian Capital Territory, Australia; 3 Queensland Brain Institute, The University of Queensland, St. Lucia, Queensland, Australia; 4 School of Information Technology and Electrical Engineering, The University of Queensland, St. Lucia, Queensland, Australia; Lund University, Sweden

## Abstract

**Background:**

While considerable scientific effort has been devoted to studying how birds navigate over long distances, relatively little is known about how targets are detected, obstacles are avoided and smooth landings are orchestrated. Here we examine how visual features in the environment, such as contrasting edges, determine where a bird will land.

**Methodology/Principal Findings:**

Landing in budgerigars (*Melopsittacus undulatus*) was investigated by training them to fly from a perch to a feeder, and video-filming their landings. The feeder was placed on a grey disc that produced a contrasting edge against a uniformly blue background. We found that the birds tended to land primarily at the edge of the disc and walk to the feeder, even though the feeder was in the middle of the disc. This suggests that the birds were using the visual contrast at the boundary of the disc to target their landings. When the grey level of the disc was varied systematically, whilst keeping the blue background constant, there was one intermediate grey level at which the budgerigar's preference for the disc boundary disappeared. The budgerigars then landed randomly all over the test surface. Even though this disc is (for humans) clearly distinguishable from the blue background, it offers very little contrast against the background, in the red and green regions of the spectrum.

**Conclusions:**

We conclude that budgerigars use visual edges to target and guide landings. Calculations of photoreceptor excitation reveal that edge detection in landing budgerigars is performed by a color-blind luminance channel that sums the signals from the red and green photoreceptors, or, alternatively, receives input from the red double-cones. This finding has close parallels to vision in honeybees and primates, where edge detection and motion perception are also largely color-blind.

## Introduction

Over the past few decades, considerable effort has been devoted to investigating how vision guides insect flight, especially in flies and bees [1]–[4]. As a result, we now have a reasonably good understanding of how flying insects regulate flight speed, avoid collisions with obstacles, negotiate narrow gaps, and orchestrate smooth landings. However, relatively little is known about how birds perform these tasks.

This study begins to address this discrepancy by examining whether, and how budgerigars use visual features to direct and guide their landings. The budgerigar (*Melopsittacus undulatus*) is a native Australian bird found mostly in inland Australia. Budgerigars are highly aerobatic, have a well developed visual system, and are known to be sensitive to the three human primary colors [5], as well as to ultraviolet light [6]. Thus, they provide an attractive model system in which to investigate visual guidance of bird flight, particularly in relation to the use of visual features in the environment, and of color. Here we investigate what visual cues guide budgerigars towards a landing site.

Earlier studies of visually guided landings in birds have concentrated on identifying the visual cues that trigger various phases of the landing maneuver. Gannets plummeting into the sea to catch fish consistently close their wings at a constant time prior to contact with the water surface, irrespective of the speed at which they approach the water or the height at which they commence their dive [7]. When a Harris hawk (*Parabuteo unicinctus*) lands on a perch, it extends its claws in preparation for landing at a constant time (τ) prior to making contact with the perch [8]. On the other hand, pigeons (*Columba livia*) show a characteristic head bobbing during landing, which is not observed in case of the hawk [9]. This head bobbing may prevent the use of τ as a factor for timing landing in the case of pigeons [10]. However, in a further study it was shown that pigeons use τ as a factor for landing under conditions of stress [11]. In a subsequent study it was shown that pigeons control braking before landing by keeping 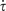, (the rate of change of τ) constant [12].

The aim of our study is to determine whether, and how, the budgerigar uses visual features to guide its landings. We find, firstly, that landings are directed primarily at regions of the scene that carry contrasting visual features, such as the edges of objects. Secondly, we find that the process of detecting the edge appears to be mediated by a “color blind” system, although the budgerigar as a whole is known to possess well-developed, tetrachromatic color vision [5], [6].

## Methods

### Ethics Statement

All experiments were carried out in accordance with the Australian Laws on the protection and welfare of laboratory animals and the approval of the Animal Experimentation Ethics Committees of the Australian National University, Canberra, Australia, and the University of Queensland, Brisbane, Australia. “This project has been reviewed and ethical clearance obtained from the University of Queensland's Animal Ethics Committee (Native and exotic wildlife and marine animals).”

### Subjects

**Figure 1:**
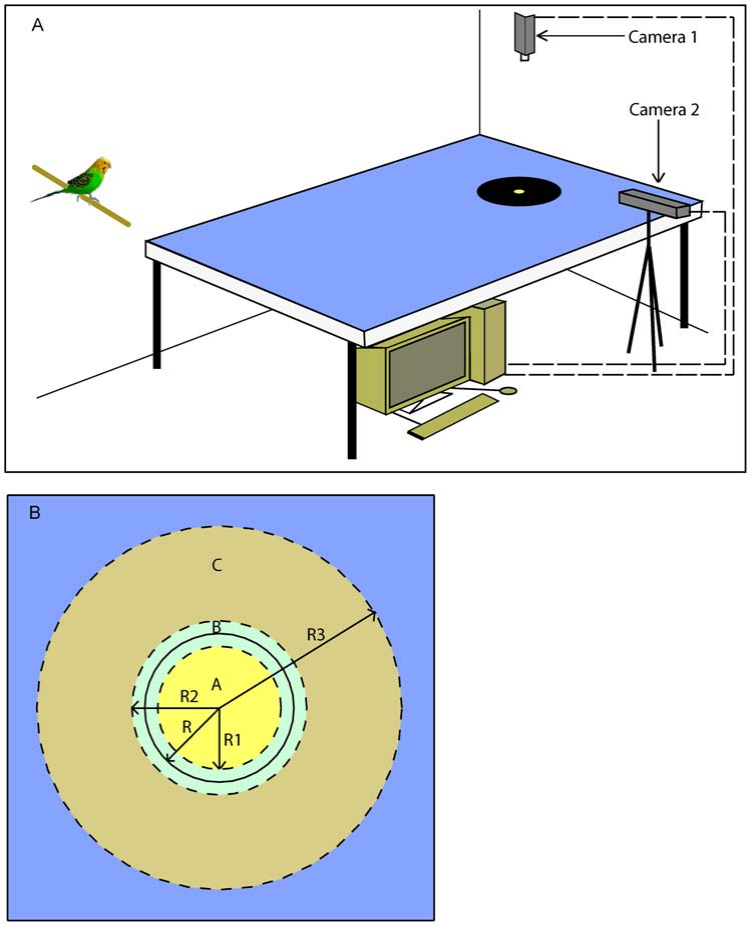
Experimental arena. (A) Budgerigars were trained in the laboratory to take off from a perch and land at a Petri dish containing bird seed, placed at the centre of a grey paper disc 41.5 cm in diameter. The disc was placed over a blue background of length 247 cm and width 256 cm. The landings were video-filmed from above and from the side. (B) Illustration of the regions A (yellow), B (blue) and C (light brown) used for the analysis of the spatial distribution of the landings. Details in “Methods”.

Adult male wild type budgerigars (n = 3−6, approximately 1 year old) served as subjects for the experiments. The birds were obtained from different local breeders. Male budgerigars were identified by a characteristically green plumage and a distinctly blue nasal coloration. The birds were housed in pairs in identical cages of length 47 cm, breadth 34.5 cm and height 82 cm, and were not under acoustic or visual isolation. All of the birds were housed indoors in a room (of length 400 cm, breadth 300 cm and height 240 cm), which also served as their training and experimental room. The room did not carry any extraneous visual landmarks. Indoor lighting was provided by means of Phillips daylight fluorescent tubes (Phillips Power Miser TLD 36 W, NSW, Australia). There were two lamps in the ceiling, with two fluorescent tubes in each lamp. The lights were controlled by an automatic timer (WF, WF-60A, Hagemeyer, UK Ltd.), which provided a 12∶12 L:D photoperiod. The lamps operated at the standard frequency of 50 Hz and therefore generated pulses of illumination at 100 Hz. The critical flicker fusion frequency (CFF) of budgerigars has been reported to be in the range of 40–75 Hz (Figure 1, [13]). The CFF is in the range of 80–105 Hz for domestic hens [14], [15], 55–105 Hz for African Grey parrots [14], and 73–140 Hz for pigeons [14], depending upon illumination levels and other factors. Therefore, it is likely that the 100 Hz fluorescent illumination used in our experiments was at or close to the budgerigars' CFF.

The illumination spectrum of the room in which the experiments were carried out was measured. The lights were controlled by an automatic timer (WF, WF-60A, Hagemeyer, UK Ltd.), which provided a 12∶12 L:D photoperiod.

Seed and water were provided ad libitum. The budgerigars were supplied with commercial budgerigar seed mix (Trill, budgerigar seed mix, Wacol, Queensland, Australia). The seed mix contained a mixture of seeds, shell grit and essential vitamins and minerals. The birds were also fed occasionally with apples and greens. Daily, the birds were moved to an adjoining screened patio of length 763 cm, breadth 203 cm and height 231 cm, where they were released from their cages and allowed to fly between perches. This enclosure provided the opportunity for regular flight as well as exposure to natural daylight. It also contained a bird bath.

### Apparatus

The experiments were carried out in the room described above, which did not carry any extraneous visual landmarks. A large horizontal surface was created by arranging nine tables (each of length 79 cm, breadth 79 cm and height 72 cm), in a 3×3 matrix. The surface of the table was covered with blue paper (Kingfisher Blue 402 275 036, Canford paper 150 gsm, Daler Rowney, Bracknell, England) (Figure 1A).

Since a single large piece of paper was not available, individual papers of A1 size were pasted breadth wise, using double-sided tape, to form a blue background of length 247 cm and breadth 256 cm). Upon this background was placed a disc of 41.5 cm diameter, of one of several grey levels ranging from black to white. The grey papers used for the discs were Jet Black (402 275 004)^1^ Mouse Grey (0741657)^2^, Sombre Grey (999960202)^2^, Dreadnought Grey (402 275 023)^1^, Azure Blue Grey (402 275 003)^1^, and Snow White (402 275 068)^1^, [^1^Canford paper, Daler Rowney, Bracknell, Berkshire, England;^2^ Canson card, Arjo Wiggins Pty.Ltd, Keysborough, Victoria, Australia].

### Training

The budgerigars were trained to fly from a wooden perch to a feeder, placed in the middle of a grey disc (Figure 1A). The feeder consisted of a transparent Petri dish of 8.7 cm diameter, containing budgerigar seed mix. For each trial a trained bird was randomly chosen and allowed to fly from the perch to the feeder. The bird was induced to take off by rotating the perch slowly. Upon landing, the bird was allowed to eat a few seeds from the Petri dish. The total duration of each trial was 5 minutes. The food reward was present in all of the trials. The reason for this was that removal of the reward destroyed the motivation of the birds to land near the previous location of the food source and caused them to land randomly anywhere on the table, or to not even leave the perch. During each trial the remaining birds were kept under visual isolation so that they were unable to observe the experimental procedure. None of the experiments involved food deprivation.

On a given day each bird was used for 10 trials on a given color card, and then kept away from the experimental room for the rest of that day. However the same bird was used for the same color card on subsequent days, again for 10 trials. Hence, for any given color card, each bird contributed 30–35 trials. Between 100 and 201 trials were performed for each card. Data from certain trials were excluded from analysis, for the reasons detailed in Table S1. 3–6 birds were used in each experiment.

The grey discs as well as the Kingfisher Blue background were replaced when they had acquired a significant number of bird droppings. This was done because the bird droppings created distracting visual features that attracted landings.

### Control experiment to test for color discrimination

For reasons that will be explained in the Results section, it was necessary to test whether the budgerigars were able to discriminate the color of the Dreadnought Grey disc from the color the Kingfisher Blue background. To this end, 4 birds were trained to receive a food reward from a Petri dish placed on the Dreadnought Grey disc, and presented over the Kingfisher Blue background. After 10 rewarded trials, the trained birds were tested by offering them a choice between two discs, one Dreadnought Grey and the other Kingfisher Blue, both placed side by side with their centers 90 cm apart over the Kingfisher Blue background (Figure S1). In the tests each disc carried a Petri dish with a food reward, but the dish was sealed with a transparent lid to prevent access to the food (This was done to avoid reinforcement during the tests.). The tests were conducted in blocks of 10 trials, with 10 further training trials inserted between successive test blocks. The spatial positions of the Dreadnought Grey disc and the Kingfisher Blue disc were swapped in consecutive test blocks (It was experimentally impractical to swap the disc positions randomly from trial to trial within a test block, because the discs had to be affixed firmly to the background to prevent edge artifacts.). In the tests, the birds flew toward the discs and landed on or close to one of them, thus displaying their choice preference. We measured the relative choice frequencies of the birds for the two test discs, to assess their ability to distinguish between the colors of Dreadnought Grey and Kingfisher Blue.

### Recording of bird landings

Landings were recorded using two synchronized video cameras (Jai Pulnix TM-9701d). One camera, attached to the ceiling of the room, filmed the landings from a position above the grey disc while the second camera filmed the lateral view of the landing area. Each camera carried a Computar TV lens with a fixed focal length of 8.5 mm (M 8513; CBC Co., Ltd, Tokyo, Japan). Both cameras captured video at 30 frames per second. The videos were directly recorded on a computer (PC, AMD Athlon) equipped with an ATA Raid controller and Euresys camera card, using software developed in-house with Visual C and Visual Basic (Microsoft Corporation, Redmond, Washington, USA).

### Analysis of video data

The video recordings were analyzed by playing back the video recordings frame by frame and digitizing the position and orientation of the bird at the point of touchdown using a Matlab (Mathworks, USA) program developed in-house. The radial distribution of landing densities was measured by counting the landings that occurred in three concentric regions in and around the disc (described below). The landing density for each region was calculated as the number of landings per unit area in that region.

The three regions were (a) an inner circle (radius (R1) = 34.4 cm), (b) an annular region containing the boundary of the disc (inner radius (R1) = 34.4 cm, outer radius (R2) = 48.6 cm) and (c) an outer annulus (inner radius (R2) = 48.6 cm, outer radius (R3) = 101.6 cm). These regions are shown in Figure 1B as A (yellow), B (light blue) and C (beige) respectively. The disc is shown as the circle with the solid boundary, of radius (R) = 41.5 cm.

The rationale for the choice of these three regions is as follows. We wished to measure and compare the numbers of landings occurring “inside” the disc and in the “boundary” region. Since landings directed at the boundary of the disc seldom occurred precisely at the edge, but within a region surrounding the boundary, we defined the “boundary region” as an annulus containing the boundary, and extending a small and equal distance on either side of it (i.e. with an inner radius R1 and an outer radius R2), and having an area equal to that of the inner circle (A) of radius R1. We defined the inner circle A to be the “inside region” of the disc, and the annulus B (of inner radius R1 and outer radius R2) to be the “boundary region” of the disc. R1 and R2 were chosen such that (i) the area of the boundary region B is equal to that of the inside region A and (ii) the boundary region extends an equal distance away from the boundary on either side of it (i.e. R2-R = R-R1). This choice of equal “inside” and “boundary” regions for the disc allowed us to make an objective comparison of the landings occurring within the disc, with the landings occurring at its boundary. If the regions A and B elicit equal numbers of landings, we can infer that the boundary of the disc is just as attractive as the interior of the disc. If B elicits a greater proportion of landings, then the boundary is more attractive; if A elicits a greater proportion, the interior is more attractive. It can be shown that the radii R1 and R2 that describe the sizes of the inner circle and the boundary annulus to satisfy the above constraints are given by R1 = 0.828R and R2 = 1.172R. For a disk of radius R = 41.5 cm (see above) we obtain R1 = 0.828R = 34.4 cm and R2 = 1.172R = 48.6 cm, as indicated above.

The radius R3 of the outermost circle was chosen to define the largest possible area over the surface of the table that excluded regions close to the boundary of the table, and other features on the walls of the room that could potentially produce interfering effects. R3 was chosen to be 101.6 cm, which was close to the edge of the table. Landings occurring outside this region were excluded from the analysis.

The landing density for each region was calculated as the number of landings per unit area in that region. From this, two measures of landing performance were obtained: (i) The *normalized landing density* for each region was calculated by dividing the landing density in that region by the total number of landings that had occurred within the entire area under consideration (i.e. within the circle of radius R3); (ii) The *landing density ratio* (α) for the boundary annulus was calculated as the ratio of the landing density in the annulus to the average landing density over the entire area under consideration.

Data, obtained with the six different grey discs and the control disc (of the same Kingfisher Blue color as the background), were analyzed using the method described above.

### Definition, measurement and calculation of contrasts

The contrast produced in each spectral class of photoreceptor was calculated as described in Lehrer et al. [16]. The procedure is summarized briefly below.

### Photoreceptor excitation

The photoreceptor excitation is given by ∫ *P*(λ).I(λ).R(λ).d(λ).

**Figure 2:**
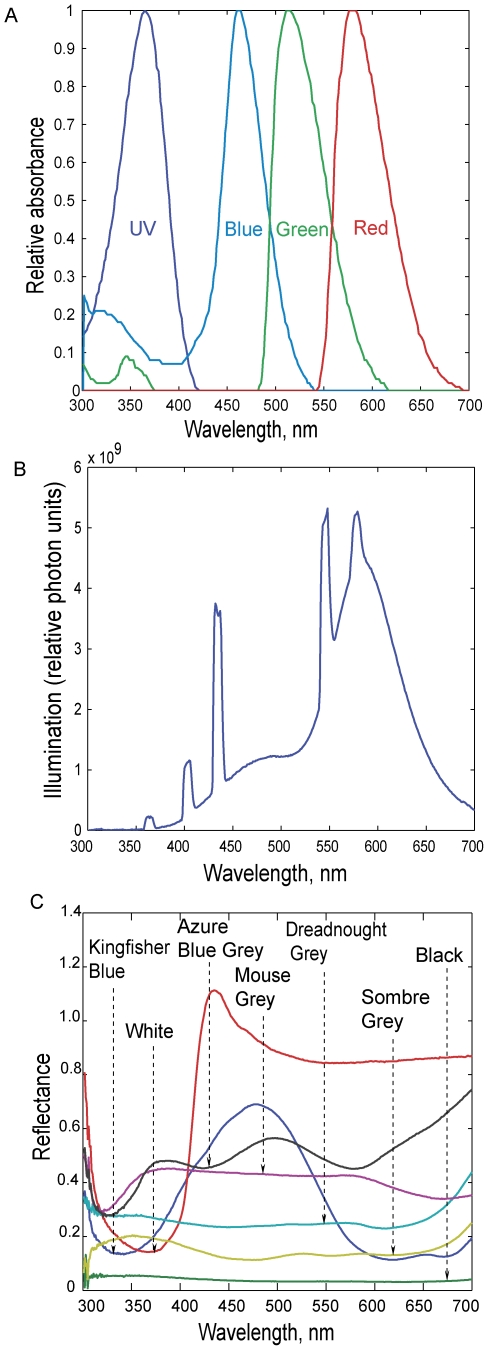
Spectral plots. (A) Absorbance spectra of the visual pigments of the Budgerigar. (B) Illumination spectrum of the room in which the experiments were carried out. (C) Reflectance spectra of the various discs used in the experiments.

In the above expression, P(λ) is the absorption spectrum of the photopigment. The absorption spectra were obtained from Goldsmith & Butler [17] by digitizing the curves in the lower panel of their Figure 2 using Digitizeit software (Digital River GmbH, Cologne, Germany). This data, sub sampled and reconstructed using linear interpolation, is shown in Figure 2A.

I(λ) is the illumination spectrum. The illumination spectrum in the experimental area was measured by pointing the probe of a calibrated fiber optic spectrometer (USB 4000 Ocean Optics Inc, Dunedin, Florida, USA) directly at one of the fluorescent lamps in the ceiling. This illumination spectrum, plotted in relative photon units, is shown in Figure 2B.

### Reflectance spectra of papers, R(λ)

The reflectance spectrum of each of the papers that was used in the experiment (all of the grey level papers, as well as the blue background) was measured by comparing the spectrum of the light reflected from the paper, P(λ), under a source of constant illumination (in this case, outdoors in the sun on a cloudless day) with the spectrum of light, S(λ), reflected from a white reflectance standard under the same illumination. The white reflectance standard possessed uniform reflectance throughout the spectral range of 330 nm–800 nm. The relative reflectance spectrum of the paper was then calculated as 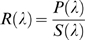. (Note that R(λ) can assume values greater than 1.0 if P((λ) is greater than S((λ) at certain wavelengths.)

P(λ) and S(λ) were measured by pointing the probe of the spectrometer at the paper (or the reflectance standard), taking care not to cast a shadow on the surface that was being measured, and that the measured surface covered the entire field of view of the probe. The measurement of each paper was preceded and followed by a measurement of the reflectance standard. The two measurements of the standard were averaged and compared with the measurement of the paper, in order to minimize any errors due to instrumental drift or varying illumination. The relative reflectance spectra of the various papers used in the experiments are shown in Figure 2C.

### Experiments

Experiments were carried out using discs of 6 different grey levels, as described above. In each case, the disc was placed on a constant Kingfisher Blue background. In addition, a control experiment was carried out in which the disc had the same color (Kingfisher Blue) as the background. This control experiment was used to check for the presence of any artifactual edges between the disc and the background.

**Figure 3:**
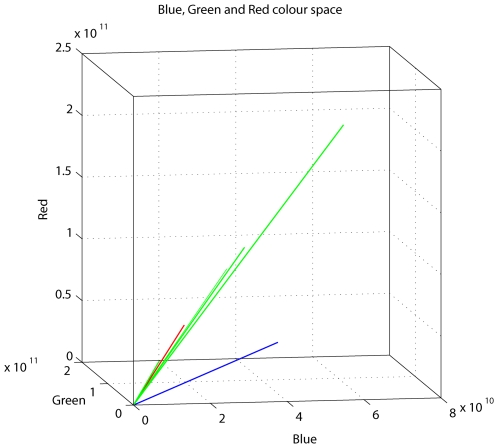
Three dimensional representations of various discs in color space. Colors of the blue background and of the various grey discs, shown as vectors representing the relative excitations of the red, green and blue photoreceptor channels. The UV excitation is not depicted, as it is very low. The blue vector represents the blue background. The green vectors represent the various grey discs, except for one grey disc (Dreadnought Grey), which is shown in red. The vectors for all of the grey discs have almost identical directions, indicating that the hues of the grey discs (as perceived by the birds) are all very similar.

Figure 3 shows the colors of the Kingfisher Blue background and of the various grey discs, as vectors representing the relative excitations of the red, green and blue photoreceptor channels. It shows that, while all of the grey cards possess the same color (the vectors are similarly oriented), the blue background has a different color, represented by a vector with a substantially different orientation.

### Statistical analysis

To quantify landing preferences, we analyzed the birds' landings on the card by measuring the density of landings within the boundary region between the disc and the background, and comparing this with the overall density of landings over all three regions (A, B and C). We define α as the ratio of the density of landings in the boundary region, to the overall landing density. Thus, a value of α = 1 would imply that birds do not prefer the boundary region at all, and land with a uniform probability density over the entire region. On the other hand, α>1 would indicate that the birds show a preference for the boundary region. The procedure used to determine if the measured value of α is different from random choice is based on the assumption that the binary choice behavior of a landing bird follows a binomial distribution. An estimate of the standard error of the mean of the distribution is given by σ = (α(1−α)/n)^1/2^ [18], [19]. In a two-tailed test, α is significantly different from the value of 1 at the P<0.05 level if α is more than 1.95σ away from 1, and at the P<0. 01 level if it is more than 2.57σ away.

## Results

Although a few birds landed directly at the Petri dish to feed, the majority landed at the boundary of the disc (i.e. in region B) and then walked to the food—even though there was no food at the boundary. Evidently, the birds were using the visual contrast that was present at the boundary to direct and guide their landings.

**Figure 4:**
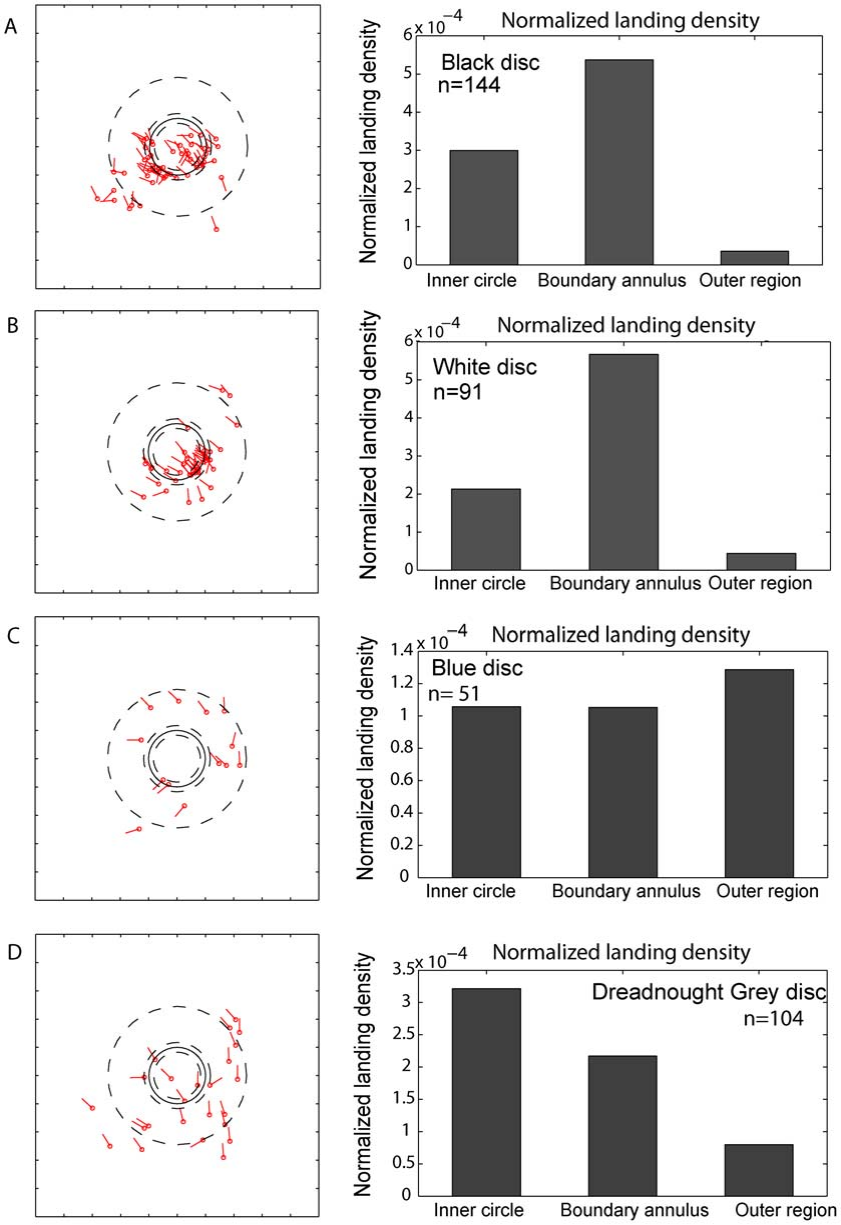
Summary of bird landings. The left hand panels show examples of the positions and orientations of landings of one bird when the disc was Snow White (A), Jet Black (B), Kingfisher Blue (C) (control) and Dreadnought Grey (D). The dot denotes the head position and the line the body orientation. The background was a constant Kingfisher blue in all cases. The right hand panels show the radial distributions of normalized landing densities for these discs in regions A, B and C (see Figure 1B). They represent a total of 390 landings from 3–6 birds.

Figure 4 shows, for one typical bird, the positions and orientations of the landings and the landing densities (number of landings per unit area) in the three regions A, B and C for four of the discs: Snow White, Jet Black, Kingfisher Blue, and Dreadnought Grey. The lines indicate the position and orientation of the body axis and the dot represents the position of the head.

This data reveals that, with the Snow White and the Jet Black discs, the highest landing density occurs in the boundary region. Thus, in each case, the boundary between the disc and the background is very effective in attracting landings. However, in the control experiment with the Kingfisher Blue disc, the landing density in the boundary region is very similar to those in the other regions, indicating that the edge between the disc and the identically-colored background is invisible to the birds. A similar result is obtained with the Dreadnought Grey disc, indicating that the boundary between this disc and the background is not very effective in eliciting landings. For all of the other grey discs (Mouse Grey, Azure Blue Grey, Sombre Grey) the boundary region elicits a higher landing density compared to the other regions (Figure S1). These results suggest that Dreadnought Grey is the only grey disc for which the boundary is nearly invisible to the landing birds.

**Figure 5:**
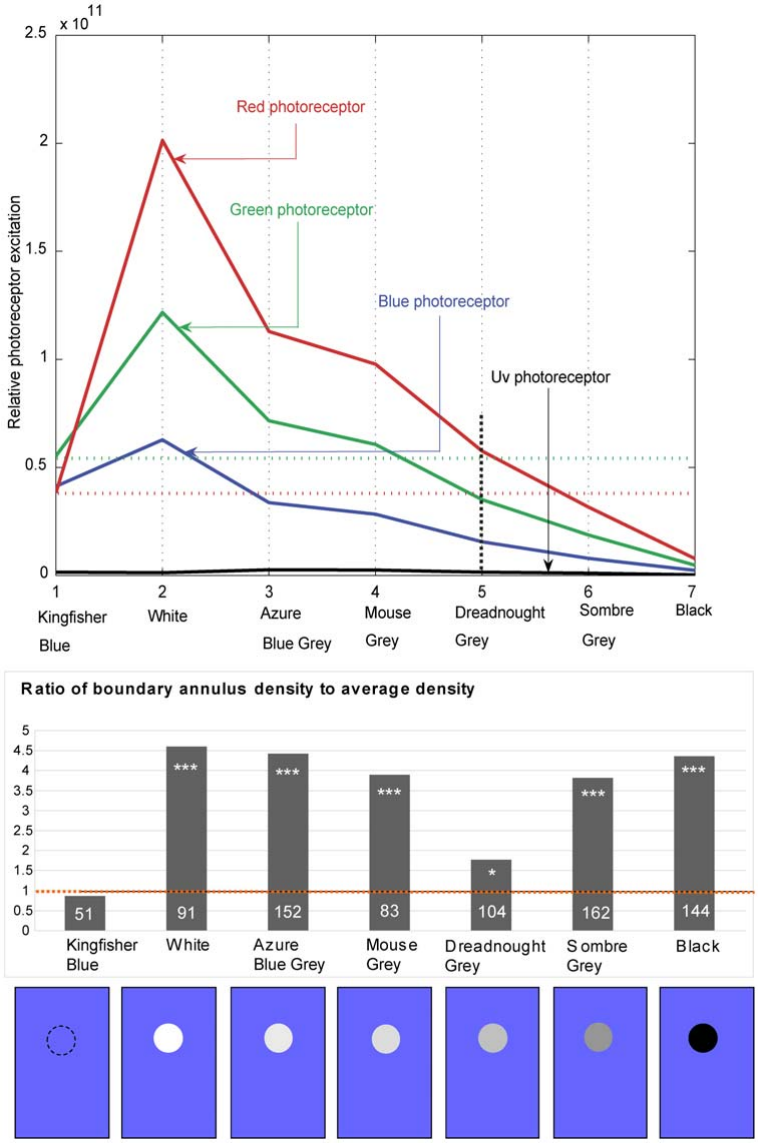
Relative photoreceptor excitations for the various color discs. Upper panel: The vertical dotted line facilitates reading of the excitations induced by the Dreadnought Grey disc in the red, green, blue and UV photoreceptors, and comparison with the excitations induced in the red and green receptors by the Kingfisher Blue background (horizontal red and green dotted lines, respectively). Lower panel: Values of α obtained for the various grey cards. α is the ratio of the density of the landings in the boundary region (region B in Figure 1B) to the average overall landing density (measured over regions A, B and C in Figure 1B). The data represent a total of 787 landings from 3–6 birds. The number in each bar denotes the number of landings analyzed. (***) indicates that the value of α is highly significantly different from 1.0 (P<0.00005), (*) indicates a marginally significant difference (0.01<P<0.05), and the absence of this symbol indicates that α is not significantly different from 1.0 (P>0.3). A pictorial representation of the various grey discs, as viewed against the blue background, is shown at the bottom of panel B.

The results for the entire data set (all experiments, all birds) are summarized in Figure 5 (lower panel). This panel shows the value of α, the ratio of the landing density in the boundary region to the overall landing density (as described in “Methods”), when the Kingfisher Blue background was held constant and the grey level of the disc was varied systematically. The value of α is highly and significantly greater than 1.0 (P<0.00005) for all of the grey discs, except for Dreadnought Grey (α = 1.76, P = 0.03). Furthermore, α for the Dreadnought Grey disc is significantly lower than that for each of the other grey discs (White, Azure Blue Grey, Mouse Grey, Sombre Grey and Black; P<0.000001 in each case; Binomial distribution z-test, [20]), and is only marginally different (P = 0.35) from that for the control disc (Kingfisher Blue). There is no significant difference between the values of α for the White, Azure Blue Grey, Mouse Grey, Sombre Grey and Black discs (P>0.09 for all pair wise comparisons). These findings reveal that there is a substantially and significantly higher density of landings in the boundary region for all of the grey discs, except for Dreadnought Grey. With the Dreadnought Grey disc the value of α was closest to 1.0, and was different from this value at only a marginally significant level, implying that in this condition the birds landed nearly randomly all over the test surface even though this grey disc is (at least for humans) clearly distinguishable from the Kingfisher Blue background (Figure 3). The contribution of each individual bird to the landing density ratio (α), and the number of landings analyzed for each bird and disc color, are given in Table S2.

The above results indicate that the disc boundary was clearly visible to the landing birds for all of the grey discs, except for Dreadnought Grey (Figure 5, lower panel). In the control experiment (Kingfisher Blue disc on an identical Kingfisher Blue background, Figure 5, lower panel) α was 0.85, which was not significantly different from 1.0 (P>0.3). This finding demonstrates that any residual visual contrast between the edge of the disc and the background had a negligible effect in eliciting landings. Therefore, the vast majority of landings that occur within the boundary region in the other experiments must be due to the presence of a perceptible visual contrast (to the birds) between the disc and the background, and not due to any artefacts at the boundary.

When the disc and the background are both Kingfisher Blue, the birds land on the visually uniform areas, but far less frequently. We find that many of these residual landings then occur completely outside the region of interest (C), or at bird droppings, seeds or small visual imperfections on the surface of the paper. Table S1 gives, for each disc color, the total number of flight trials conducted, and the number of trials excluded from the analysis for various reasons, as explained in the table. It is clear that the percentage of these excluded trials is substantially larger when the disc is Kingfisher Blue (i.e. the same color as the background), or Dreadnought Grey (little or no edge contrast). Under each of these conditions, the birds show an increased tendency to land either completely outside region C, or at bird droppings or visual imperfections. Furthermore, Table S1 shows a reciprocal relationship between the visibility of the disc boundary, and the tendency to land at spurious features or at locations outside region C. These findings further support our conclusion that landings are guided principally by visually contrasting features.

**Figure 6:**
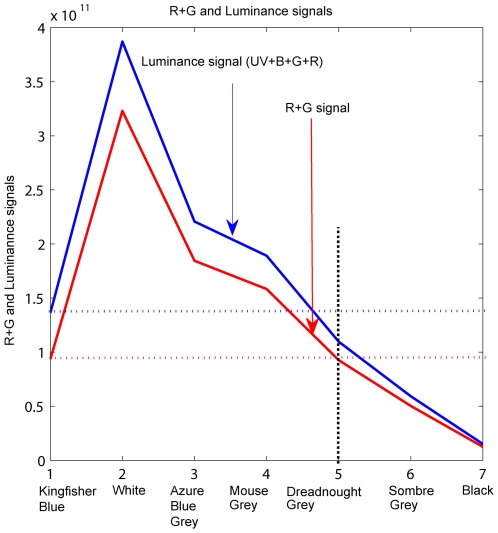
Relationship between the luminance signals for the various colored discs. Variation of the luminance signal (UV+B+G+R) and the (R+G) signal for the various colored discs, calculated as described in “Methods”. The vertical dotted line facilitates reading of the (UV+B+G+R) signal and the (R+G) signal induced by the Dreadnought Grey disc, and comparison with the corresponding signals induced by the Kingfisher Blue background (horizontal blue and red dotted lines, respectively).

The relative photoreceptor excitations produced by the various grey cards in the red, green, blue and UV photoreceptors in the retina of the budgerigar were computed as described in the “Methods” section (Figure 5, upper panel). When the disc is Dreadnought Grey, we see from Figure 5 (upper panel) that the red photoreceptor receives approximately the same excitation from the disc (0.55) as it does from the blue background (0.4). The same is true for the green photoreceptor, which receives excitations of 0.55 from the Kingfisher Blue background and 0.4 from the disc. This means that with the Dreadnought Grey disc on the Kingfisher Blue background, neither the red receptor nor the green receptor experiences a strong contrast at the boundary. However, neither the red receptor nor the green receptor alone exhibits a perfect match of excitations from the Kingfisher Blue background and the Dreadnought Grey disc. On the other hand, the sum of the excitations of the red and green receptors produces a perfect match (Figure 6). We also note that a “total luminance” signal, comprising the sum of the UV, blue, green and red signals, produces a poorer match (Figure 6). Thus, if we postulate that edge detection for landing is mediated by a ‘color-blind’ visual subsystem that receives input from a sum of the signals from the red and green receptors; we have an explanation for why the birds behave as though they barely detect the boundary between the disc and the background when the disc is Dreadnought Grey.

A control experiment was conducted to examine whether the birds could distinguish between the color of the Dreadnought Grey disc and the color of the Kingfisher Blue background. Four birds, trained on the Dreadnought Grey disc as described in the “Methods” section, subsequently chose the Dreadnought Grey disc (over the blue disc) 50 times in 60 test trials (Figure S2). The behavior of the trained birds in the tests did not show any evidence of spatial memory playing a role in their choices. At the start of each test block, the trained birds immediately flew to the correct disc, even though it was now in a different position compared to the previous test block. The trained birds' preference for the Dreadnought Grey disc was statistically highly significant (P<0.00005, using the binomial statistics described in “Methods”). This demonstrates that, although the visual subsystem that guides the budgerigar's landings does not detect the boundary between the Dreadnought Grey disc and the Kingfisher Blue background, the bird's color vision system is clearly capable of distinguishing between these two colors.

## Discussion

It is known that, during long-range migration, pigeons (*Columba livia*) use visual landscape features comprising lines (such as roads) or edges (such as the shores of lakes, or the boundaries of fields or forests) as navigational aids [21]. Here, we have shown that edges play an important role in directing and guiding landings. Since a visually contrasting edge is likely to represent the edge of an object, it would be a favorable place to land, as it would offer the bird's claws a good grip at the point of touchdown. Thus, it would seem advantageous to direct landings at contrasting edges; and we can conclude that the principle of “affordance”, as espoused originally by Gibson [22] is used by birds to seek suitable locations for landing. Our findings further suggest that the visual subsystem that detects edges and guides landings is color-blind, and could possibly be a visual modality that predates the evolution of color vision. The ability to detect edges almost disappears when the Dreadnought Grey disc is presented against the Kingfisher Blue background (Figures 4, 5, lower panel). The reason for the weak residual preference for the boundary region may be that the Dreadnought Grey disc does not offer precisely the level of grey at which the visibility of the boundary disappears.

We see from Figure 5 (upper panel) that, with the Dreadnought Grey disc, the excitation produced by the disc is similar to that produced by the background, for the red as well as the green receptors. A perfect match of the excitations that are produced by disc and the background is obtained if we postulate that edge detection is performed by a color-blind pathway that sums the red and the green signals.

Color-blindness in edge detection and motion perception has also been observed in honeybees [16], which possess excellent trichromatic color vision comprising UV, blue and green photoreceptors. There, landings appear to be guided by a visual subsystem that is driven exclusively by the green photoreceptors. Movement detection in honeybees is also color blind, and is driven by the green photoreceptors [23].

Since the Dreadnought Grey disc and the Kingfisher Blue background disc possess very different colors (see Figure 3), these colors must be easily discriminated by the bird's color vision system. Dual-choice training experiments reveal that budgerigars can indeed distinguish between these two colors readily (Figure S2). Nevertheless, the edge detection system that guides landing is evidently driven by a color-blind signal that is incapable of this color discrimination.

The parallel observations in the budgerigar and the bee suggest that the ability to use color vision to distinguish between objects, but the inability to use color information to detect edges, may be a common feature of many flying species. Budgerigars carry the so-called red “double cone” photoreceptors, which constitute 50% of the total population of cone receptors in the retina. The absence of an oil droplet in one of the double cones endows this type of photoreceptor with a spectral sensitivity that is somewhat broader than that of a single red photoreceptor with an oil droplet [17]. This makes the spectral sensitivity of the red double-cone photoreceptor similar to that of a system that pools signals from the red and green photoreceptors. Thus, our findings suggest that the visual subsystem that mediates edge detection during landing is driven by a color-blind system that pools signals from the red and green photoreceptors, or, alternatively, derives its input exclusively from the red, double-cone photoreceptors. Our experiments do not allow us to distinguish between these two possibilities. If the edge-detecting system were to pool the red and green signals, it would be analogous to the “luminance” channel in the primate visual system, which is color-blind and known to be involved in the perception of movement [24]. On the other hand, if the edge-detection system is driven by the red double cone photoreceptors, then it is possible that the red double cones constitute the luminance channel in birds, and mediate edge detection as well as motion perception. Given the dominant presence of the red double cones in the bird retina, and the importance of accurate landing to survival, this intriguing possibility deserves to be explored.

## Supporting Information

Table S1 Composition of data, showing total flight trials conducted for each disc color, the numbers of landings excluded from analysis for various reasons, and the number of landings analyzed.
(0.04 MB DOC)

Table S2 Summary of landing density ratios (α) for the middle annulus for different birds on various discs, with the number of landings analyzed in each case shown in parentheses. When the number of landings in a particular condition is zero (meaning that the particular bird and disc were not tested), α is designated ‘not applicable’ (n/a).
(0.04 MB DOC)

Figure S1 Summary of bird landings. The left hand panels show examples of the distributions of landings of one bird when the disc was Mouse Grey (A), Azure Blue Grey (B), and Sombre Grey (C). The dot denotes the head position and the line the body orientation. The background was a constant Kingfisher Blue in all cases. The right hand panels show the radial distributions of landing densities for these discs. They represent a total of 397 landings from 3–6 birds.
(1.29 MB TIF)

Figure S2 Results of color discrimination control experiment. Four birds, trained on the Dreadnought Grey disc as described in the “Methods” section, subsequently chose the Dreadnought Grey disc (over the Kingfisher Blue disc) 50 times in 60 test trials.
(0.95 MB TIF)

Video S1 The video shows a budgerigar landing on the edge of a Jet Black disc placed on a uniform Kingfisher Blue background.
(0.18 MB MOV)
