# Supplementary material for: Edge Detection in Landing Budgerigars (Melopsittacus undulatus)
Source: PLoS One. 2009 Oct 7;4(10):e7301. doi: 10.1371/journal.pone.0007301 (PMC2752810; doi:10.1371/journal.pone.0007301)
Supplement: Table S1 — Composition of data, showing total flight trials conducted for each disc color, the numbers of landings excluded from analysis for various reasons, and the number of landings analyzed. (0.04 MB DOC) [file pone.0007301.s001.doc]

**Table S1** Composition of data, showing total flight trials conducted for each disc color, the numbers of landings excluded from analysis for various reasons, and the number of landings analyzed.

| Disc color | Total flight  trials | Total flights  analyzed | Total flights excluded | Data excluded due to landings occurring outside region C | Landings discounted due to bird droppings, seeds and visual imperfections | Data lost due to video file corruption |
| --- | --- | --- | --- | --- | --- | --- |
| Kingfisher Blue | 100 | 51 | 49 (49%) | 46 | 2 | 1 |
| White | 100 | 91 | 9 (9%) | 9 | 0 | 0 |
| Azure Blue | 201 | 152 | 49 (24%) | 34 | 11 | 4 |
| Mouse Grey | 100 | 83 | 17 (17%) | 14 | 2 | 1 |
| Dreadnought Grey | 201 | 104 | 97 (48%) | 75 | 20 | 2 |
| Sombre Grey | 200 | 162 | 36 (18%) | 29 | 7 | 0 |
| Black | 200 | 144 | 56 (28%) | 44 | 11 | 1 |
