# Supplementary material for: Edge Detection in Landing Budgerigars (Melopsittacus undulatus)
Source: PLoS One. 2009 Oct 7;4(10):e7301. doi: 10.1371/journal.pone.0007301 (PMC2752810; doi:10.1371/journal.pone.0007301)
Supplement: Table S2 — Summary of landing density ratios (α) for the middle annulus for different birds on various discs, with the number of landings analyzed in each case shown in parentheses. When the number of landings in a particular condition is zero (meaning that the particular bird and disc were not tested), α is designated ‘not applicable’ (n/a). (0.04 MB DOC) [file pone.0007301.s002.doc]

**Table S2** Summary of landing density ratios (α) for the middle annulus for different birds on various discs, with the number of landings analyzed in each case shown in parentheses. When the number of landings in a particular condition is zero (meaning that the particular bird and disc were not tested), α is designated ‘not applicable’ (n/a).

| Disc color | Kingfisher Blue | White | Azure Blue Grey | Mouse Grey | Dreadnought Grey | Sombre Grey | Black |
| --- | --- | --- | --- | --- | --- | --- | --- |
| Bird Name |
| Google | 0.41  (21) | 4.93  (30) | 5.28  (66) | 6.31  (29) | 2.3  (53) | 4.24  (80) | 4.22  (66) |
| Drongo | 1.45  (12) | 4.71  (37) | 4.81  (38) | 2.29  (19) | 1.74  (15) | 2.84  (49) | 5.0  (47) |
| Budgie | 1.09  (26) | 3.99  (24) | 3.48  (30) | 3.3  (29) | 0.48  (18) | 4.35  (32) | 3.65  (31) |
| Acer | n/a  (0) | n/a  (0) | 4.35  (2) | 0.0  (2) | 1.58  (11) | 0.0  (1) | n/a  (0) |
| Casper | n/a  (0) | n/a  (0) | 2.18  (8) | 0.0  (3) | 1.74  (5) | n/a  (0) | n/a  (0) |
| Icarus | 0.0  (2) | n/a  (0) | 1.09  (8) | 0.0  (1) | 0.0  (2) | n/a  (0) | n/a  (0) |
| Pooled data | 0.85  (51) | 4.59  (91) | 4.41  (152) | 3.88  (83) | 1.76  (104) | 3.82  (162) | 4.35  (144) |
