# Supplementary figures and images for: Edge Detection in Landing Budgerigars (Melopsittacus undulatus)
Source: PLoS One. 2009 Oct 7;4(10):e7301. doi: 10.1371/journal.pone.0007301 (PMC2752810; doi:10.1371/journal.pone.0007301)

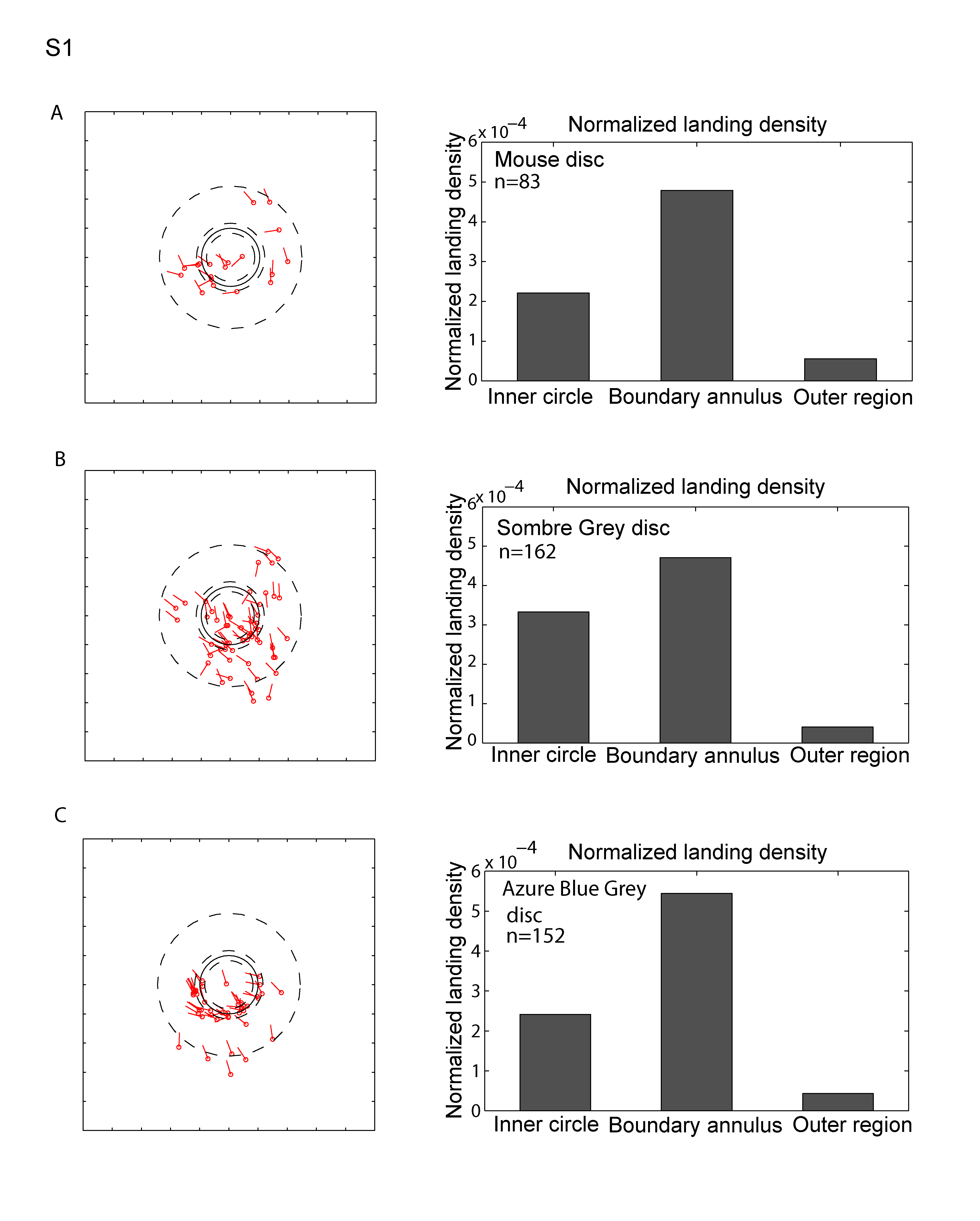

Supplement: Figure S1 — Summary of bird landings. The left hand panels show examples of the distributions of landings of one bird when the disc was Mouse Grey (A), Azure Blue Grey (B), and Sombre Grey (C). The dot denotes the head position and the line the body orientation. The background was a constant Kingfisher Blue in all cases. The right hand panels show the radial distributions of landing densities for these discs. They represent a total of 397 landings from 3–6 birds. (1.29 MB TIF) [file pone.0007301.s003.tif]

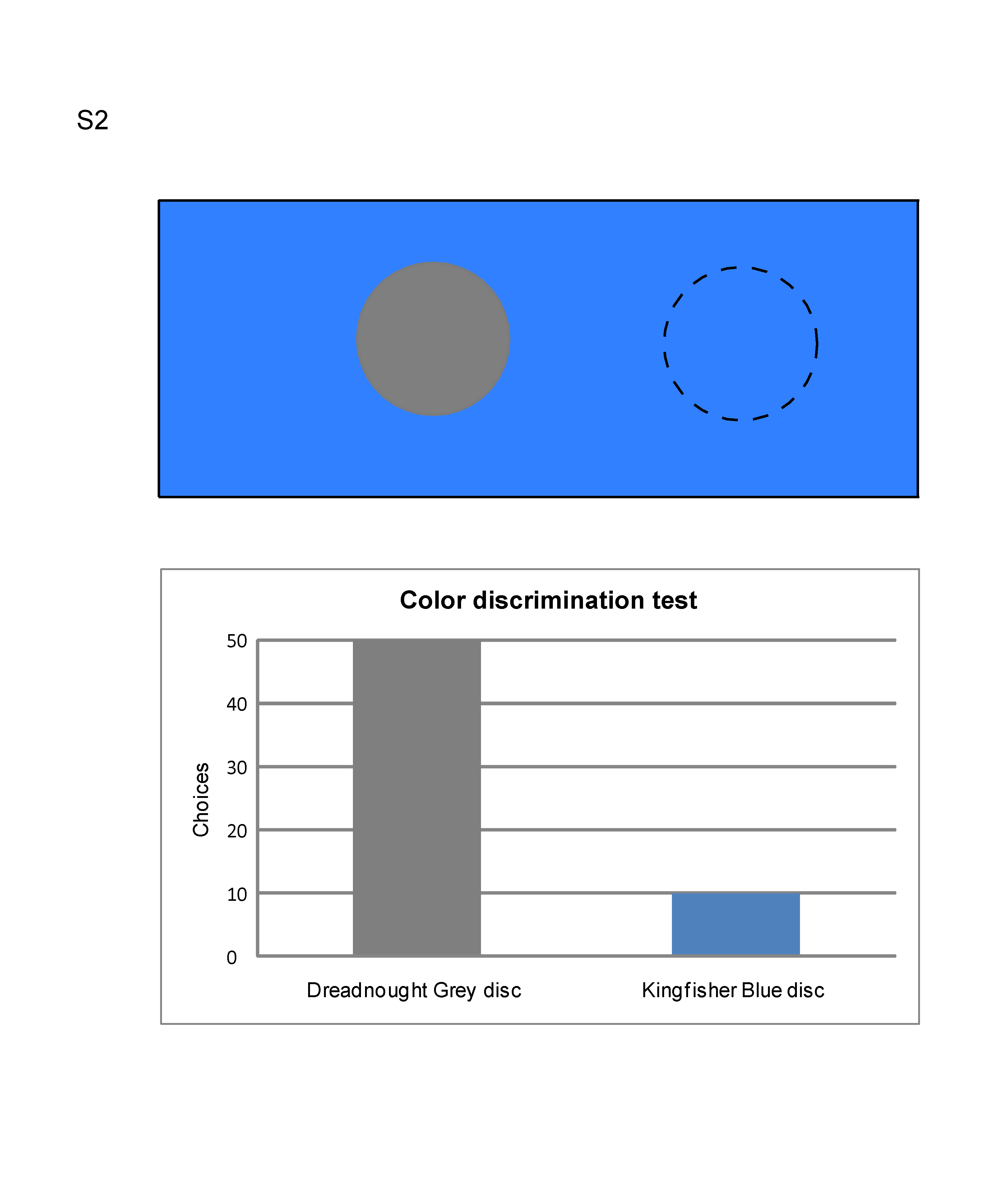

Supplement: Figure S2 — Results of color discrimination control experiment. Four birds, trained on the Dreadnought Grey disc as described in the “Methods” section, subsequently chose the Dreadnought Grey disc (over the Kingfisher Blue disc) 50 times in 60 test trials. (0.95 MB TIF) [file pone.0007301.s004.tif]
